# Experimental Demonstration of a Stacked Hybrid Optoacoustic-Piezoelectric Transducer for Localized Heating and Enhanced Cavitation

**DOI:** 10.3390/mi12101268

**Published:** 2021-10-18

**Authors:** Pil Gyu Sang, Deblina Biswas, Seung Jin Lee, Sang Min Won, Donghee Son, Jong G. Ok, Hui Joon Park, Hyoung Won Baac

**Affiliations:** 1 Department of Electrical and Computer Engineering, Sungkyunkwan University, Suwon 16419, Korea; pgsang@skku.edu (P.G.S.); deblina@skku.edu (D.B.); sjleee@skku.edu (S.J.L.); sangminwon@skku.edu (S.M.W.); daniel3600@g.skku.edu (D.S.); 2 Department of Mechanical and Automotive Engineering, Seoul National University of Science and Technology, Seoul 01811, Korea; jgok@seoultech.ac.kr; 3 Department of Organic and Nano Engineering, Hanyang University, Seoul 04763, Korea; huijoon@hanyang.ac.kr

**Keywords:** laser-generated focused ultrasound, pulsed cavitation, tissue therapy, precision therapy

## Abstract

Laser-generated focused ultrasound (LGFU) is an emerging modality for cavitation-based therapy. However, focal pressure amplitudes by LGFU alone to achieve pulsed cavitation are often lacking as a treatment depth increases. This requires a higher pressure from a transmitter surface and more laser energies that even approach to a damage threshold of transmitter. To mitigate the requirement for LGFU-induced cavitation, we propose LGFU configurations with a locally heated focal zone using an additional high-intensity focused ultrasound (HIFU) transmitter. After confirming heat-induced cavitation enhancement using two separate transmitters, we then developed a stacked hybrid optoacoustic-piezoelectric transmitter, which is a unique configuration made by coating an optoacoustic layer directly onto a piezoelectric substrate. This shared curvature design has great practical advantage without requiring the complex alignment of two focal zones. Moreover, this enabled the amplification of cavitation bubble density by 18.5-fold compared to the LGFU operation alone. Finally, the feasibility of tissue fragmentation was confirmed through a tissue-mimicking gel, using the combination of LGFU and HIFU (not via a stacked structure). We expect that the stacked transmitter can be effectively used for stronger and faster tissue fragmentation than the LGFU transmitter alone.

## 1. Introduction

Focused ultrasound (FUS) with high frequencies (a few tens of MHz) and high amplitudes capable of producing acoustic cavitation (tens of MPa) has been generated using an optoacoustic effect [1]. Nanosecond laser pulses (e.g., 5–10 ns) have enabled the excitation of thin-film optoacoustic transmitters, e.g., a composite film of carbon nanotube (CNT) and polydimethylsiloxane (PDMS) [2,3,4,5]. An optoacoustic lens with a transmitter layer coated on a spherically concave substrate has been fabricated to produce powerful FUS with high-frequency and broad bandwidth [1,6,7,8,9]. Such laser-generated focused ultrasound (LGFU) emitted from the optoacoustic lens produces non-thermal and micro-cavitational disturbances at a tight focal spot (<100 µm), which has been utilized for micro-precision treatment such as tissue fragmentation (i.e., histotripsy), cellular membrane opening for molecular delivery, thrombolysis, and targeted cell detachment/disconnection [10,11,12,13].

In such cavitational treatment, a negative peak pressure amplitude of US pulse should reach a certain threshold to initiate acoustic cavitation. For a given medium, the probability of cavitation generation also depends on temperature, acoustic frequency, and initial gas content. Nominal values for peak pressure amplitudes in properly degassed and room-temperature environments have been reported as −26 MPa in deionized water, −15.4 MPa in fatty tissue, −6 MPa in blood, and −19.2 MPa in muscle [14,15,16]. Considering the intrinsic attenuation of each medium, which increases with the penetration depth, a stronger pressure amplitude than such thresholds is necessary from a transmitter surface. This is to overcome such attenuation and then exceed the threshold for cavitation at a target depth. LGFU operated in a high center frequency of 13~16 MHz (a 6 dB roll-off point at approximately ~30 MHz) is especially even more susceptible to attenuation than other US cases in a low MHz frequency range.

However, the maximum LGFU amplitude is always limited for a given optoacoustic lens by the physical damage threshold of the transmitter material under pulsed laser irradiation. Due to the limited LGFU amplitude, the penetration depth for cavitational treatment has been limited to a few-to-several mm thus far [1,10,11,12,13,17]. This demands an alternative way to produce LGFU-induced cavitation, for example, by employing an additional transducer whose focal point is superposed [13,18]. Previously, a dual-focusing scheme was introduced in which LGFU with a tight focal spot of 100 µm is overlapped with an additional pressure pulse focused by a piezoelectric transducer (800 µm in focal width) [18]. This superposition configuration allows the amplitude summation of two pressure pulses without considering any thermal effect at focus. The focal pressure amplitude to reach the cavitation threshold by LGFU alone could be reduced to some extent this way.

The cavitation process can be thermally facilitated, as the nucleation of vapor phase in liquid can be triggered by thermal fluctuations [19,20]. This significantly increases the cavitation probability for a given volume and a treatment time. Thus, such thermal facilitation to form cavitation is an effective way since the threshold requirement can be mitigated [21,22]. With a single piezoelectric transducer, this heating approach has been applied to generate bubbles in boiling histotripsy [23,24,25,26]. However, if LGFU is used alone, it is extremely difficult to cause significant heat deposition by itself due to the low pulse repetition rate (typically, a few to 20 Hz; limited by laser systems) whose pulse-to-pulse interval is much longer than heat diffusion time over a microscale focal zone (e.g., in water or tissues).

We demonstrate LGFU (center frequency ~16 MHz) with a heated focal zone formed by HIFU. First, thermal enhancement of LGFU-induced cavitation was characterized using a superposition alignment of two separate focal transmitters in which two FUS transmitters (i.e., HIFU and LGFU) were arranged perpendicular to each other (i.e., with an angle difference between two FUS axes *θ* = 90°). HIFU-induced temperature enhancement at the focal zone from 22 up to 35 °C in water increased the rate of LGFU-induced cavitation significantly from 6% to 36% by an input laser energy as low as 4.35 mJ/pulse. Based on the above feasibility of enhanced cavitation, we developed a longitudinally superposed configuration (*θ* = 0°) by fabricating a stacked hybrid transducer where an optoacoustic transmitter layer was coated directly on the piezoelectric HIFU transmitter. This allowed the alignment of two foci to be readily formed by fabrication by sharing a single curvature, thus significantly resolving the alignment complexity and making a standalone operation of the single combined device. Importantly, free-boundary cavitation in water was obtained by the enhanced LGFU performance by HIFU-induced heating, which is an essential step to move forward for tissue therapy (e.g., micro-histotripsy). Finally, fragmentation of a tissue-mimicking gel was performed using a disintegrated arrangement of two FUS (*θ* = 180°) whose focal zone shape was almost identical with that of the stacked configuration (*θ* = 0°). The hybrid structure transmitting both LGFU and HIFU from the single shared curvature would be practically useful for high-precision cavitation therapy, allowing thermal and non-thermal treatment simultaneously.

## 2. Materials and Methods

**Figure 1:**
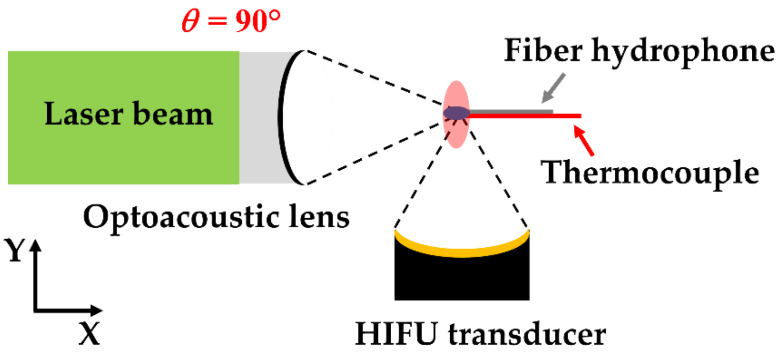
Experimental setup showing the superposition of LGFU and HIFU (*θ* = 90°); the focal zone of LGFU shown with the blue ellipse directed along the *x*-axis, and the focal zone of HIFU with the red ellipse along the *y*-axis).

Figure 1 illustrates the experimental schematic for the dual-focusing configuration of LGFU and HIFU. Two different transducers were placed with *θ* = 90°. This configuration was primarily used to study the effect of HIFU-induced heating on cavitation generation by LGFU. We fabricated an optoacoustic lens (40 mm in diameter and with a 26 mm radius of curvature; *f*-number = 0.65) by depositing a CNT-PDMS composite film on top of a concave-shaped glass substrate to produce LGFU. Firstly, a solution of multi-walled CNTs and toluene (1:100 ratio) was prepared and sonicated for 1 h for homogenized mixing. Then, the CNT–toluene solution was coated on a concave glass substrate by a spray method. The prepolymer liquid and curing agent (10:1 ratio) were mixed to prepare a uniform PDMS solution. Then, this PDMS solution was drop-casted on the CNT layer. Finally, the CNT–PDMS composite layer on the concave substrate was cured at 90 °C for 2 h. Subsequently, an Nd:YAG pulsed laser beam (Litron Lasers Ltd., Rugby, Warwickshire, United Kingdom. 532 nm wavelength, 7 ns width, and 10 Hz pulse repetition rate) was used for excitation of the CNT–PDMS-coated lens which had axial and lateral focal widths (6 dB) that were 280 and 85 µm, respectively. We used an HIFU generator (Dongiltech, Republic of Korea, model number 15B021) as an additional transmitter (22 mm outer diameter, 20 mm diameter of ceramic disk, 18 mm radius of curvature, and *f*-number = 0.9) to form a heated zone greater than the focus of LGFU, which had axial and lateral focal spot widths of 3.5 mm and 0.62 mm. A sinusoidal signal (2 V peak-to-peak and 4 MHz frequency) initially generated by a function generator (Rigol 1022A, Handelsweg, Germany) and amplified by an RF amplifier (5082FE, OPHIR, Los Angeles, United States) was used for driving the HIFU transducer. The focal points of both transmitters were aligned for spatial superposition by using a single-mode fiber-optic hydrophone as illustrated in Figure 1 [27,28]. The initiation timing of two US waves was synchronized for temporal superposition. Then, the duty cycle of the sinusoidal signal was set to 20% and 60% to create the temperature increase of 9 and 13 °C, respectively, at the focus measured by using a k-type thermocouple. Both transmitters were embedded in a water tank with an ambient temperature of 21~22 °C.

**Figure 2:**
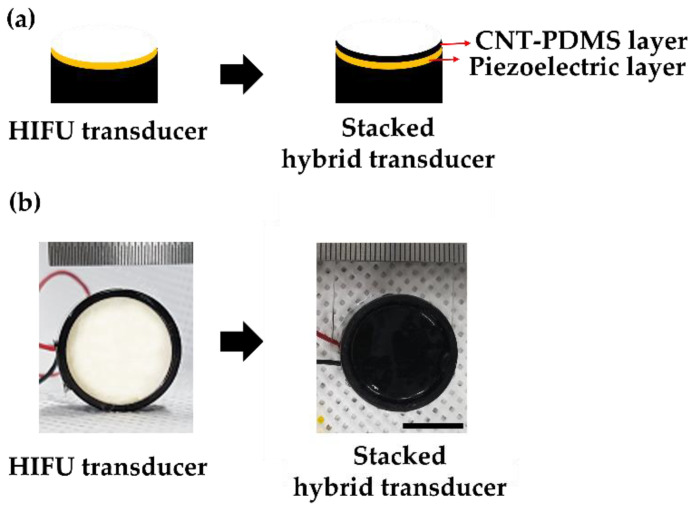
(**a**) A cross-sectional schematic of the stacked hybrid transducer. The yellow layer indicates the piezoelectric transmitter for HIFU. The top black layer presents the CNT–PDMS film which was directly coated upon the piezoelectric transmitter. (**b**) Photographs (**top** view) of the piezoelectric transmitter (**left**) and the fabricated hybrid transmitter with the CNT–PDMS layer (**right**). (Scale bar = 10 mm).

As the superposition alignment shown in Figure 1 is a laborious and time-consuming process, requiring a high spatial accuracy for two separate focal systems, we developed a stacked hybrid transducer for simultaneous generation of LGFU and HIFU from a single shared curvature. For fabrication, an optoacoustic transmitter layer was coated directly onto a piezoelectric HIFU transducer. This combined configuration resolved the alignment complication, as two focal spots were aligned readily by the single spherical substrate. This certainly enhanced the accuracy of focal spot superimposition and reduced time required for precise alignment of two different transducers. For the stacked hybrid transducer, a CNT–PDMS composite film with a few tens of micrometer in thickness was spray-coated on the surface of a prefabricated HIFU transducer with a 20 mm diameter and 18 mm radius of curvature (*f*-number = 0.9; center frequency = 4 MHz). Then, the composite layer on the HIFU transducer was cured for 2 h at 90 °C. Figure 2 shows the stacked hybrid transducer with the bottom piezoelectric layer and the top CNT PDMS layer, both of which had an identical diameter and a radius of curvature defined by the substrate.

**Figure 3:**
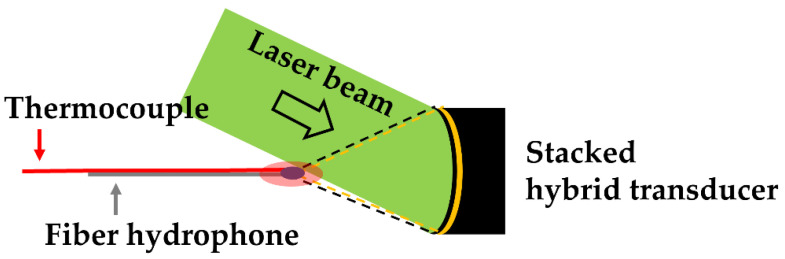
A schematic arrangement for dual-focused US from the stacked hybrid transducer (*θ* = 0°). It is shown with the input laser beam for optoacoustic generation. The focal zone of the LGFU is shown with the small blue ellipse. The greater red ellipse indicates the focal zone of the HIFU.

Figure 3 illustrates the configuration for generating the dual-focused US from the stacked hybrid transducer. The nanosecond pulsed-laser beam was used for optoacoustic generation with a slanted angle of 45°. The HIFU transducer was driven by a RF-amplified sinusoidal signal (2 V peak-to-peak and 4 MHz frequency, 20% duty cycle) from a function generator. The dual-focused US waves obtained from this configuration were measured using the fiber-optic hydrophone aligned at the focal spot. The temperature of the focal zone was also monitored using the k-type thermocouple. Free-boundary cavitation bubbles were observed using a laser shadowgraphy system [29,30].

## 3. Results and Discussion

The major objective of this study was to develop transmitter configurations that can lower the input laser energy for generating acoustic cavitation by enhancing the temperature of the focal zone for therapeutic applications. The first step to achieving this goal is to characterize thermal enhancement of cavitation generation. In this regard, we used the dual-focus configuration with *θ* = 90°.

**Figure 4:**
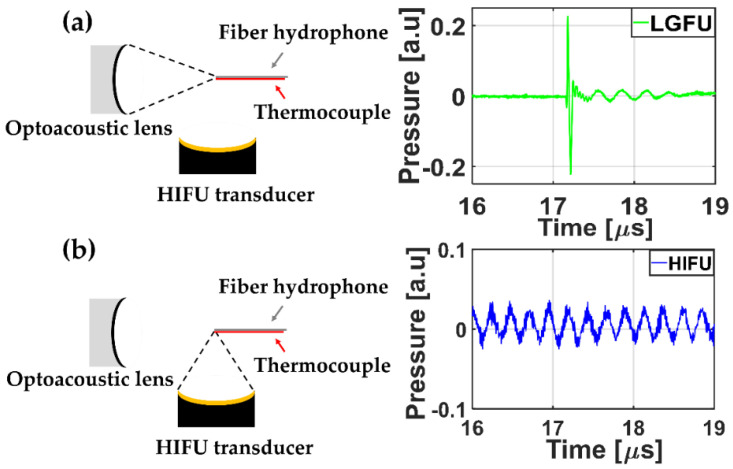
Temporal waveforms detected by a single-mode fiber-optic hydrophone when only the LGFU was turned on (**a**) and only when the HIFU was turned on (**b**).

**Figure 5:**
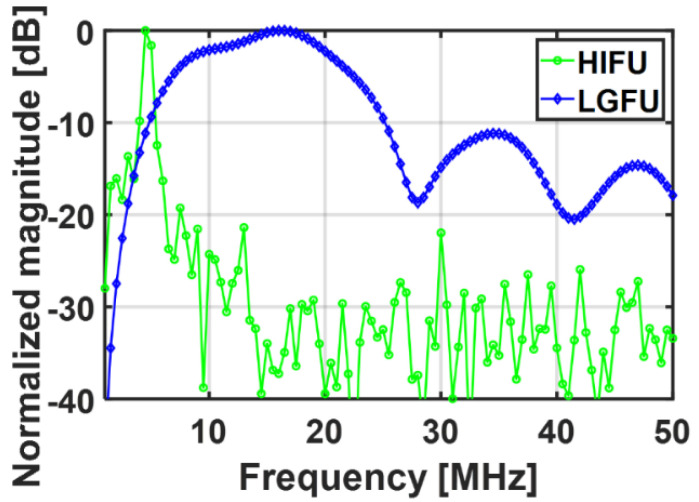
Normalized frequency spectra of the HIFU and the LGFU (the detector bandwidth effect was included for both cases).

A temporal profile of each FUS was acquired by using the fiber-optic hydrophone. Figure 4 shows the measured US waveforms. While the temporal profile of the HIFU transducer alone (Figure 4b) illustrated a continuous sinusoidal waveform, the temporal profile of the LGFU (Figure 4a) showed a narrow-width bipolar waveform (input laser energy = 4.35 mJ/pulse). Such a pulse shape with the short temporal width was proportional to the time derivative of input laser pulse, which was observed typically in optoacoustic generation with a far-field configuration. The normalized magnitude along frequency spectra is shown in Figure 5. The central frequencies of the HIFU and the LGFU were 4.5 and 16 MHz, respectively. The 6 dB cut off on the high-frequency edge was observed at 5 MHz for the HIFU and 23.5 MHz for the LGFU. This indicates that the LGFU exhibited a broadband and high-frequency spectrum compared to the HIFU. For both cases, no cavitation signal was detected at the focal zone.

**Figure 6:**
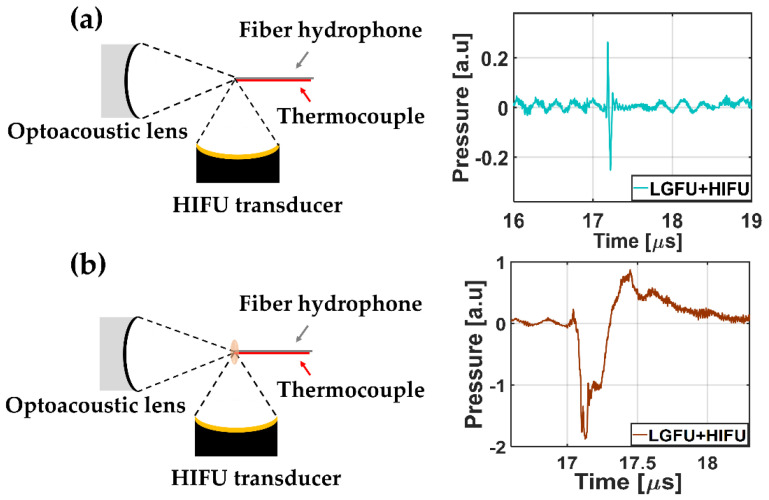
Combined waveforms of the LGFU and the HIFU are shown with negligible temperature enhancement in (**a**) (less than a few °C) and 9 °C enhancement in (**b**). While peak amplitudes of the LGFU and the HIFU were fixed for both figures, the duty cycle of the HIFU transducer was applied differently. For both operations in (**a**,**b**), temporal waveforms were recorded using the fiber-optic hydrophone after the temperature measurement.

Then, both transmitters were turned on, synchronized, and overlapped in space and time as shown in Figure 6. The combined US waveform (from Figure 4a,b) is shown in Figure 6a. In this case, the HIFU transducer turned on very shortly not to cause significant temperature enhancement (less than a few °C), resulting in the simple non-thermal addition effect of two US amplitudes. Subsequently, in Figure 6b, the duty cycle of the function generator for the HIFU increased (~20%) to raise the temperature of the overlapped focus region. The temperature increased by 9 °C. Although there was no change in the peak amplitude of the HIFU, the acoustic cavitation signal was detected clearly (Figure 6b) with strong disturbance, following the rarefactional phase of the LGFU waveform. This result confirms that cavitation can be achieved by the heated focal zone formed by the HIFU transducer, although the LGFU amplitude alone is not sufficient to produce acoustic cavitation. This was further confirmed by enhancing the focal zone temperature to 13 °C by changing the duty cycle (~60%) of the HIFU transducer. Strong acoustic cavitation was observed due to the temperature enhancement of the focal zone from 9 to 13 °C. With the 13 °C enhancement, we observed a stronger cavitation signal with an enhanced peak amplitude and a longer lifetime than the 9 °C enhancement case (not shown here). These cavitation signals were subsequently utilized to count the cavitation occurrence.

**Figure 7:**
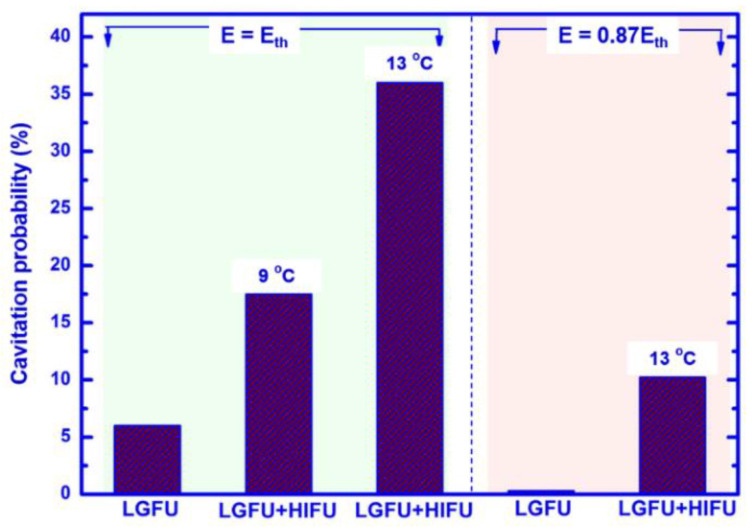
Cavitation occurrence due to the input laser energies of 4.35 mJ/pulse and 3.78 mJ/pulse.

Next, we compared cavitation probabilities for different LGFU and HIFU conditions (Figure 7). The probability was obtained by dividing the number of cavitation occurrence at focus by the number of input laser pulses. LGFU was produced by the optoacoustic lens with an input laser pulse energy of *E*. Here, *E_th_* is defined as a single-pulse laser energy to generate the focal pressure near the onset of cavitation (not the cavitation threshold of 50% probability). The operation with LGFU alone produced cavitation with a 6.1% probability (98 out of 1600 pulses) at *E* = *E_th_* which was 4.35 mJ/pulse. The chances of cavitation occurrence significantly increased by using the dual focusing of LGFU and HIFU. As the focal zone of the LGFU increased by 9 °C, the cavitation probability significantly improved to 17.5% (140 out of 800 pulses). The focal zone of the LGFU was completely included by that of the additional low-frequency HIFU with the 83-fold greater volume. This was further enhanced up to 36% (288 out of 800 pulses) in the 13 °C enhancement condition. These results also suggest that even a lower laser energy than *E_th_* can be used to produce cavitation as long as the focal zone is sufficiently heated. For this purpose, we reduced the input laser energy to 3.78 mJ/pulse which corresponds to a sub-threshold regime for the original value of *E_th_*. With this laser energy, the LGFU alone did not produce any cavitation, resulting in 0%. However, after increasing the temperature of the focal zone by 13 °C, again by the additional HIFU, the probability significantly increased to 10.3% (82 out of 800 pulses). This generated the cavitation better than the LGFU alone, previously excited with 15% higher laser energy (*E* = 4.35 mJ/pulse) resulting in the onset level (6.1%). Hence, this is evident from this study that LGFU-induced cavitation can be facilitated by the heated focal zone and can even be achieved at the sub-threshold regime in terms of the laser energy required to produce cavitation.

**Figure 8:**
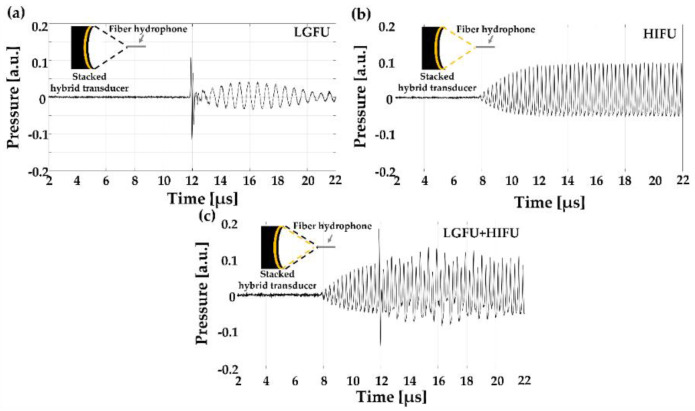
FUS waveforms generated from the stacked hybrid transducer: (**a**) LGFU alone; (**b**) HIFU alone; (**c**) LGFU and HIFU together.

Based on the feasibility of thermal enhancement of LGFU-induced cavitation, we employed a stacked hybrid transmitter for standalone operation of two FUS from a single-shared curvature. The time–domain waveforms from the hybrid transmitter were acquired at the focal zone. Figure 8a illustrates the bipolar LGFU waveform at ~12 µs (i.e., corresponding to the radius of curvature (~18 mm)) obtained solely by pulsed-laser irradiation (10 mJ/pulse) without turning on the HIFU transmitter. The ringing effect after the LGFU pulse resulted from the backing substrate including the piezoelectric layer. Similarly, the HIFU waveform (20% duty cycle) without LGFU (no laser irradiation) was measured in the same manner (Figure 8b). The maximum pressure amplitude for HIFU was also obtained at ~12 µs. Then, the transmitter surface was irradiated with the pulsed-laser beam together with the application of an amplified sinusoidal waveform for HIFU. Figure 8c illustrates the time–domain waveform generated by the spatio-temporal superposition of LGFU and HIFU turned on simultaneously.

**Figure 9:**
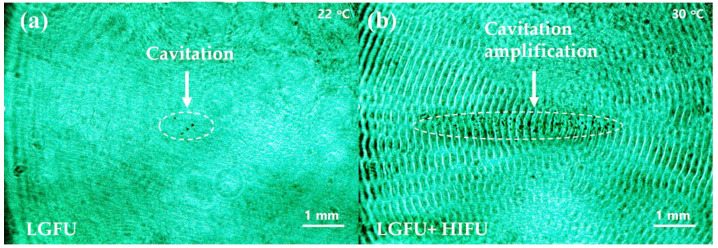
Laser shadowgraphy images for free-boundary cavitation in water: (**a**) a few cavitation bubbles are shown in free boundary in water, generated by LGFU alone; (**b**) by HIFU-induced heating (8 °C), the LGFU-induced cavitation in free boundary was greatly enhanced. The cavitation bubbles are shown over a broad range within the white dotted ellipses.

The input laser energy was increased up to 120 mJ/pulse without causing any damage to the stacked transmitter (i.e., damage threshold energy > ~120 mJ/pulse). We used such maximum-input laser energy in an effort to check whether LGFU alone can produce free-boundary cavitation. Figure 9a shows a few micro-bubbles obtained in a free-field condition in water. We note that LGFU alone can produce the free-boundary cavitation which is an essential step to move forward for biomedical treatment. Although our stacked transmitter can generate free-field cavitation, this was achieved near the upper limit of input laser energy. This means there was almost no margin to further increase the input energy and thus enhance the output pressure and cavitation. It would be difficult to utilize the LGFU-induced cavitation obtained in the above manner for practical treatment conditions likely having more acoustic attenuation mechanisms than in water. With the identical optoacoustic excitation condition for LGFU used in Figure 9a, we turned on the HIFU transmitter simultaneously in Figure 9b to induce localized heating over a region greater than the focal volume of LGFU and then boost the free-boundary cavitation effect. The white dotted region in Figure 9b clearly depicts the amplified cavitation effect with the temperature enhancement from 22 to 30 °C. The density of cavitation bubbles increased drastically from 3778 to 70,084 mm^−3^, i.e., an 18.5 times enhancement by the heat-induced boost effect. This amplified cavitation suggests that the combined configuration may possibly be employed for enhanced tissue fragmentation or faster cavitational treatment by the larger damage zone.

Despite the compact design and the capability of free-boundary cavitation generation, the focal pressure from the current stacked structure was geometrically limited due to the *f*-number of the spherical curvature (~0.9) fixed by the piezoelectric substrate. We note that optoacoustic lenses previously reported have been designed and fabricated easily a with lower *f*-number values (e.g., 0.61) [17]. The focal gain of the current LGFU from the stacked transducer was estimated to be 169, which is lower than 220 in the previous design with an *f*-number of 0.61. Moreover, the short focal distance of the current setup can block the part of pulsed-laser irradiation that is directed from the top side onto the CNT–PDMS layer. As we used the slanted angle for optoacoustic excitation, some portion of the laser beam was shadowed by the rim of transducer, thus reducing the irradiation efficiency. These issues may be resolved by employing an HIFU transmitter with a lower *f*-number, larger aperture dimension, and a longer focal length.

**Figure 10:**
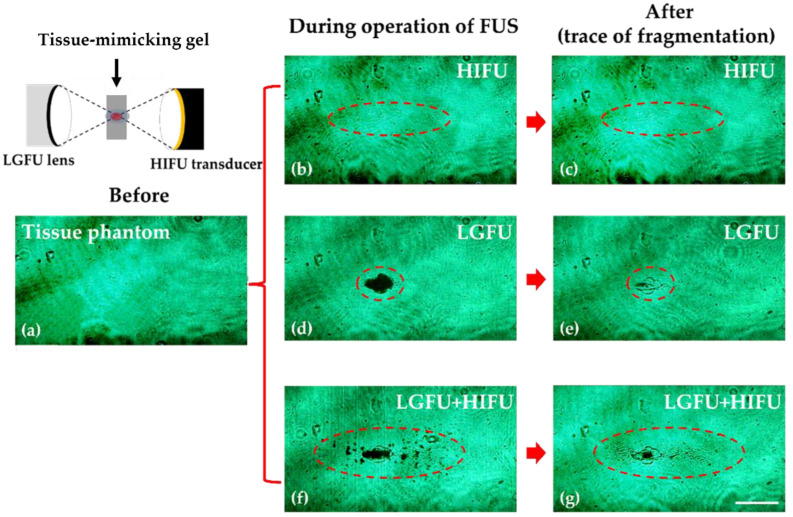
Shadowgraphy images of tissue phantom that was fragmentated by an LGFU-HIFU face-to-face (*θ* = 180°) configuration. (**a**) Agarose gel tissue phantom before applying LGFU and/or HIFU. (**b**) Real-time FUS operation with only HIFU and (**c**) the traces of fragmentation after only HIFU operation. (**d**) Real-time cavitation in tissue phantom with only LGFU operation and (**e**) the fragmentation traces after LGFU operation. (**f**) Cavitation in tissue phantom with LGFU and HIFU in real-time and (**g**) the fragmentation traces after LGFU and HIFU operation. When operating both the LGFU and HIFU at the same time, cavitation was significantly more intense than operating only the LGFU or HIFU. The lateral spot dimensions of LGFU and LGFU+HIFU were 236 and 394 µm, respectively. The axial spot dimensions of LGFU and LGFU+HIFU were 338 and 1302 µm, respectively. (Scale bar = 500 µm).

In order to ascertain the tissue fragmentation capability by the dual-focus mode, we employed an anti-parallel arrangement of the LGFU and HIFU (*θ* = 180°) as illustrated in Figure 10. The focal zone shape formed by this configuration was similar than that of the stacked configuration (*θ* = 0°). This configuration makes an anti-parallel overlap of two focal zones in which their propagation directions are axially opposite. Cavitation-induced fragmentation was performed using the tissue-mimicking agarose gel at a depth of ~15 mm. The HIFU and LGFU were operated with a duty cycle of 20% and a laser energy of 120 mJ/pulse, respectively. Figure 10 shows the laser shadowgraphy images for cavitational fragmentation resulted in real time during FUS treatment (Figure 10b,d,f) and after treatment (Figure 10c,e,g). Figure 10b confirms that the HIFU alone, inducing the mild heat deposition from 22 to 30 °C, did not produce cavitation bubbles. Here, our heating condition for the focal zone was far below the denaturization temperature of agarose gel. Figure 10d shows that the cavitation within the tissue phantom was produced solely by the LGFU. The black spot in Figure 10d, depicting the LGFU-induced cavitation region, was slightly greater than the fragmented spot after treatment as shown in Figure 10e.

Figure 10f,g illustrate the fragmentation result by the superposition of LGFU and HIFU, clearly confirming the stronger fragmentation effect. Figure 10f shows that the primary zone for cavitational disturbance appeared at the tightly focused spot defined by LGFU, which was similar in the case of Figure 10d. However, many cavitation bubbles were also observed along the HIFU focal zone and was much greater than that of LGFU. As the LGFU amplitude was significantly lower than the 6 dB of peak amplitude of the LGFU, these scattered cavitation bubbles in Figure 10f led to relatively weak traces after fragmentation as shown in Figure 10g.

The anti-parallel arrangement in Figure 10 shows the feasibility for cavitation-induced therapy by our hybrid configuration for LGFU and HIFU. However, this also reveals that the LGFU amplitude from the stacked structure was relatively limited by the shallow piezoelectric curvature with the high *f*-number of 0.9. The LGFU amplitude should be further enhanced to utilize the stacked structure for therapy applications in a standalone manner. This requires an HIFU transmitter designed with a low *f*-number or a large aperture dimension to achieve a high focal gain for LGFU. Otherwise, the optical irradiation arrangement or a laser beam profile projected on the transmitter’s surface should be improved to increase optical absorption [31].

## 4. Summary

We demonstrated the dual-focusing configurations of LGFU and HIFU to boost the LGFU-induced cavitation effect via mild heat deposition obtained by the HIFU transmitter. The thermal facilitation of LGFU-induced cavitation was characterized by the spatio-temporal superposition of two different FUS waves: LGFU (16 MHz frequency) and HIFU (4.5 MHz frequency). The rate of cavitation generation in the detector boundary could be significantly increased (36%). In addition, the input laser energy required to induce cavitation (*E_th_*) could be mitigated by 13% via mild heating of 13 °C. Then, a stacked single-element transmitter for both LGFU and HIFU was fabricated and utilized to resolve the alignment issue of two FUS waves, thus allowing a standalone operation of the single compact device. This stacked transmitter enabled the great enhancement of the LGFU-induced free-boundary cavitation effect by 18.5-fold in terms of the volume density of the cavitation bubbles with the heated focal zone compared to LGFU alone. However, the current stacked transmitter had limitations such as the geometrically defined focal gain and the laser irradiation arrangement. For ascertaining the tissue fragmentation capability using our combined configuration, we adopted the anti-parallel configuration in two FUS wave propagations, which was chosen to take the advantage of high focal gain of the optoacoustic lens (*f*-number ~0.65). Such a combined anti-parallel arrangement confirmed the feasibility for therapeutic applications by fragmentation of the agarose gel at the treatment depth of ~15 mm. While the primary cavitational disturbance zone was formed by strong LGFU amplitudes, the overall damage zone by the heat-induced cavitation (394 µm in the lateral direction and 1302 µm in the axial direction) was greatly increased compared to the primary zone obtained by LGFU alone (236 µm (lateral) and 338 µm (axial)). We expect that the proposed stacked transmitter can be used for tissue fragmentation at long penetration depths or in high acoustic attenuation environments.

## Data Availability

Not applicable.

## References

[1] Baac H.W., Ok J.G., Maxwell A., Lee K.-T., Chen Y.-C., Hart A.J., Xu Z., Yoon E., Guo L.J. (2012). Carbon-nanotube optoacoustic lens for focused ultrasound generation and high-precision targeted therapy. Sci. Rep..

[2] Baac H.W., Ok J.G., Park H.J., Ling T., Chen S.L., Hart A.J., Guo L.J. (2010). Carbon nanotube composite optoacoustic transmitters for strong and high frequency ultrasound generation. Appl. Phys. Lett..

[3] Lee T., Baac H.W., Li Q., Guo L.J. (2018). Efficient photoacoustic conversion in optical nanomaterials and composites. Adv. Opt. Mater..

[4] Colchester R.J., Mosse C.A., Bhachu D.S., Bear J.C., Carmalt C.J., Parkin I.P., Treeby B.E., Papakonstantinou I., Desjardins A.E. (2014). Laser-generated ultrasound with optical fibres using functionalized carbon nanotube composite coatings. Appl. Phys. Lett..

[5] Noimark S., Colchester R.J., Blackburn B.J., Zhang E.Z., Alles E.J., Ourselin S., Beard P.C., Papakonstantinou I., Parkin P., Desjardins A.E. (2016). Carbon-nanotube-PDMS composite coatings on optical fibers for all-optical ultrasound imaging. Adv. Funct. Mater..

[6] Chan W., Hies T., Ohl C.-D. (2016). Laser-generated focused ultrasound for arbitrary waveforms. Appl. Phys. Lett..

[7] Li Q., Zhu H., Feng C., He Z., Dong W., Yu H. (2019). Simple yet universal fabrication strategy for a focused photoacoustic transmitter. Opt. Lett..

[8] Aytac-Kipergil E., Alles E.J., Pauw H.C., Karia J., Noimark S., Desjardins A.E. (2019). Versatile and scalable fabrication method for laser-generated focused ultrasound transducers. Opt. Lett..

[9] Joo M.G., Lee K.-T., Sang P., Heo J., Park H.J., Baac H.W. (2019). Laser-generated focused ultrasound transmitters with frequency-tuned outputs over sub-10-MHz range. Appl. Phys. Lett..

[10] Baac H.W., Frampton J., Ok J.G., Takayama S., Guo L.J. (2013). Localized micro-scale disruption of cells using laser-generated focused ultrasound. J. Biophotonics.

[11] Baac H.W., Lee T., Guo L.J. (2013). Micro-ultrasonic cleaving of cell clusters by laser-generated focused ultrasound and its mechanisms. Biomed. Opt. Express.

[12] Di J., Kim J., Hu Q., Jiang X., Gu Z. (2015). Spatiotemporal drug delivery using laser-generated-focused ultrasound system. J. Control. Release.

[13] Kim J., Chang W.-Y., Lindsey B.D., Dayton P.A., Dai X., Stavas J.M., Jiang X. Laser-generated focused ultrasound transducers for microbubble-mediated, dual-excitation sonothrombolysis. Proceedings of the Ultrasonics Symposium (IUS), 2016 IEEE International.

[14] Vlaisavljevich E., Lin K.-W., Maxwell A., Warnez M.T., Mancia L., Singh R., Putnam A.J., Fowlkes B., Johnsen E., Cain C. (2015). Effects of ultrasound frequency and tissue stiffness on the histotripsy intrinsic threshold for cavitation. Ultrasound Med. Biol..

[15] Maxwell A.D., Cain C.A., Duryea A.P., Yuan L., Gurm H.S., Xu Z. (2009). Noninvasive thrombolysis using pulsed ultrasound cavitation therapy–histotripsy. Ultrasound Med. Biol..

[16] Maxwell A.D., Cain C.A., Hall T.L., Fowlkes J.B., Xu Z. (2013). Probability of cavitation for single ultrasound pulses applied to tissues and tissue-mimicking materials. Ultrasound Med. Biol..

[17] Lee T., Ok J.G., Guo L.J., Baac H.W. (2016). Low f-number photoacoustic lens for tight ultrasonic focusing and free-field micro-cavitation in water. Appl. Phys. Lett..

[18] Baac H.W., Lee T., Ok J.G., Hall T., Guo L.J. (2013). Dual-frequency focused ultrasound using optoacoustic and piezoelectric transmitters for single-pulsed free-field cavitation in water. Appl. Phys. Lett..

[19] Herbert E., Balibar S., Caupin F. (2006). Cavitation pressure in water. Phys. Rev. E.

[20] Caupin F., Herbert E. (2006). Cavitation in water: A review. Comptes Rendus Phys..

[21] Coussios C., Farny C., Ter Haar G., Roy R. (2007). Role of acoustic cavitation in the delivery and monitoring of cancer treatment by high-intensity focused ultrasound (HIFU). Int. J. Hyperther..

[22] Farny C.H., Holt R.G., Roy R.A. (2010). The correlation between bubble-enhanced hifu heating and cavitation power. IEEE Trans. Biomed. Eng..

[23] Maxwell A.D., Khokhlova T.D., Schade G.R., Wang Y.-N., Kreider W., Yuldashev P., Simon J.C., Sapozhnikov O.A., Farr N., Partanen A. (2014). Boiling histotripsy: A noninvasive method for mechanical tissue disintegration. J. Acoust. Soc. Am..

[24] Wang Y.N., Khokhlova T., Bailey M., Hwang J.H., Khokhlova V. (2013). Histological and biochemical analysis of mechanical and thermal bioeffects in boiling histotripsy lesions induced by high intensity focused ultrasound. Ultrasound Med. Biol..

[25] Pahk K.J., Gélat P., Sinden D., Dhar D.K., Saffari N. (2017). Numerical and experimental study of mechanisms involved in boiling histotripsy. Ultrasound Med Biol.

[26] Pahk K.J., Gélat P., Kim H., Saffari N. (2018). Bubble dynamics in boiling histotripsy. Ultrasound Med. Biol..

[27] Parsons J.E., Cain C.A., Fowlkes J.B. (2006). Cost-effective assembly of a basic fiber-optic hydrophone for measurement of high-amplitude therapeutic ultrasound fields. J. Acoust. Soc. Am..

[28] Ma J., Zhao M., Huang X., Bae H., Chen Y., Yu M. (2016). Low cost, high performance white-light fiber-optic hydrophone system with a trackable working point. Opt. Express.

[29] Lee T., Baac H.W., Ok J.G., Youn H.S., Guo L.J. (2015). Nozzle-free liquid microjetting via homogeneous bubble nucleation. Phys. Rev. Appl..

[30] Kudo N. (2015). A simple technique for visualizing ultrasound fields without Schlieren optics. Ultrasound Med. Biol..

[31] Aytac-Kipergil E., Desjardins A.E., Treeby B.E., Noimark S., Parkin I.P., Alles E.J. (2021). Modelling and measurement of laser-generated focused ultrasound: Can interventional transducers achieve therapeutic effects?. J. Acoust. Soc. Am..

